# On tower and checkerboard neural network architectures for gene expression inference

**DOI:** 10.1186/s12864-020-06821-6

**Published:** 2020-12-16

**Authors:** Vladimír Kunc, Jiří Kléma

**Affiliations:** Department of Computer Science, Karlovo náměstí 13, Prague, 121 35 Czech Republic

**Keywords:** Neural network, Tower architecture, Gene expression, Checkerboard architecture

## Abstract

**Background:**

One possible approach how to economically facilitate gene expression profiling is to use the L1000 platform which measures the expression of ∼1,000 landmark genes and uses a computational method to infer the expression of another ∼10,000 genes. One such method for the gene expression inference is a D–GEX which employs neural networks.

**Results:**

We propose two novel D–GEX architectures that significantly improve the quality of the inference by increasing the capacity of a network without any increase in the number of trained parameters. The architectures partition the network into individual towers. Our best proposed architecture — a checkerboard architecture with a skip connection and five towers — together with minor changes in the training protocol improves the average mean absolute error of the inference from 0.134 to 0.128.

**Conclusions:**

Our proposed approach increases the gene expression inference accuracy without increasing the number of weights of the model and thus without increasing the memory footprint of the model that is limiting its usage.

## Background

Determining gene expression is valuable for various medical and biological researches (e.g., [1–5],); however in spite of significant price drop in the last decade, gene expression profiling is still expensive for large scale experiments. One of the approaches lowering the costs and allowing larger-scale experiments is represented by the LINCS^1^ program which developed the L1000 platform based on Luminex bead technology. The L1000 platform measures the expression profile of ∼1,000 carefully selected *landmark genes* and then reconstructs the full gene profile of ∼10,000*target genes* [6] which is much cheaper than measuring the full expression profile directly. The inference of the full gene expression profile from the expression of the landmark genes was originally based on linear regression and then improved by a deep learning approach called D–GEX [7]. The original D–GEX is a pair of two artificial neural networks (NNs) that are able to, in contrast to the linear regression, reconstruct the non-linear patterns.

Inferring the full profile is a large-scale machine learning task that is computationally challenging as the target dimension is much higher than the input dimension [7]. A novel model based on the original D–GEX further improved the quality of the inference by introducing a novel family of adaptive activation functions called transformative adaptive activation functions (TAAFs) that allowed significantly lower error [8].

However, the model with TAAFs [8] still uses the same architecture as the original D–GEX with the exception of the adaptive activation functions. We present a novel model based on a NN with a novel architecture that further improves gene inference by replacing the dense layers with a more complex structure while not increasing the number of parameters and thus retaining the same memory constraints that are necessary for training the model on GPUs. This paper introduces two essential architecture changes that improve gene expression inference — replacing the three interconnected dense layers of original D–GEX by smaller dense units connected in a tower or checkerboard pattern and adding skip connections.

Artificial neural networks are a powerful tool and lead to state-of-the-art results in various fields such as computer vision (e.g., image classification and segmentation), speech recognition and machine translation. They are also commonly used in biology [9–11]. Our work builds on the D–GEX [7] and its extension with TAAFs [8].

### D–GEX

The original D–GEX is actually a family of nine, similar architectures; a D–GEX network consists of one to three densely connected hidden layers with 3,000, 6,000, or 9,000 neurons in each layer with the hyperbolic tangent as the activation function [7]. Due to technical constraints, the D–GEX approach consists of two networks of given architecture each inferring half of the target genes such that each network fits into the memory of the GPU used for training. The original D–GEX also included a dropout layer with rate either 0%, 10%, or 25% for better generalization leading to 27 different networks. Most of the presented D–GEX networks significantly outperformed the linear regression that was used by the LINCS program for the gene expression inference and k-nearest neighbors (KNN) regression [7].

The extension of the D–GEX [8] significantly improves the gene expression inference firstly by replacing the hyperbolic tangent activation functions with the logistical sigmoids and secondly by including novel transformative adaptive activation functions (TAAFs) which add four additional parameters per neuron that control the scale and translation of the inner activation function (i.e., the sigmoid function). These parameters increase the total number of parameters of the network only slightly as most of a NN’s parameters are the weights of the connections; furthermore, the D–GEX with TAAFs outperform the original D–GEX even when the number of neurons in each layer is set such that the total number of parameters is same for both the original and the modified D–GEX variants. The family of extended D–GEXs with TAAFs is, however, otherwise without any other changes as the same three-layered architectures were used to show that the performance gains are due to the TAAFs.

### Neural network architectures with parallel connections

Neural networks are often not as homogeneous as the D–GEX networks and often include multiple parallel connections or units that are not directly connected. One of the simplest parallel architectures is the so-called *multi-column deep neural network* (MCDNN) [12, 13] which is actually an ensemble of individual "columns" which are separately trained NNs. Other approaches include adding units that are composed of several parallel tracks which might even differ in the number of layers — e.g., the Inception modules and its variants [14] for image classification. An important architecture with parallel connection is represented by the ResNet family of networks [15] with a residual skip connection which adds the output of a layer to the output of the layer above. Both described approaches are still being researched and resulted in networks such as *Inception-ResNet* [16] and *DenseNet* [17].

An approach similar to MCDNN is represented by parallel circuits (PC) [18, 19] which partition a network into several, parallel columns of hidden layers that are not connected to each other. The PCs were developed mainly to reach weight reduction for speeding up the computations. Since PCs share an input and an output layer, the weight reduction occurs only when the network has two or more hidden layers [19]. The networks with PCs were tested on five datasets from the UCI machine learning repository [20]; however, the experiments were done using only a CPU, and thus only small networks were tested — namely with 100 and 1,000 neurons. The sparsity introduced by the PCs acted as a regularizer and helped to reduce overfitting [19].

## Methods

We have followed a similar experimental protocol as in [8] — we have used the TAAFs and the same data (including the partitioning into subsets for training, validation, and testing); thus the results are directly comparable.

### Data

The data from the Affymetrix microarray platform were used to train the networks for inferring the gene expression profile from the landmark genes. We have used very same data as used for training the D–GEX with TAAFs, the details about the data preprocessing are available in [8]. The data consists of 126,102 gene expression profiles; each is containing the expression of 942 landmark genes and 9,518 target genes. The training set includes 88,256 profiles, the validation set of 18,895 profiles and the testing set of 18,951 profiles. The training set was used directly for training the network, the validation set for parameter selection and optimization process and the testing set was used for reporting the performance and comparison of different networks as it provides an independent measure.

### Network architectures

The main contribution of this work is the introduction of a novel architecture for gene expression inference, which leads to significant improvements in the quality of the inference. The baseline model is a modification of D–GEX with TAAFs [8] which consists of three hidden, densely connected layers with 10,000 neurons in each layer — the largest D–GEX architecture consisted of only 9,000 neurons in each layer [7] but adding more neurons has proved beneficial — and an output layer. Each neuron contains the TAAF with a sigmoid as the inner activation function as in [8]; each hidden layer is with 25% dropout.

#### Tower architecture (T–D–GEX)

**Fig. 1:**
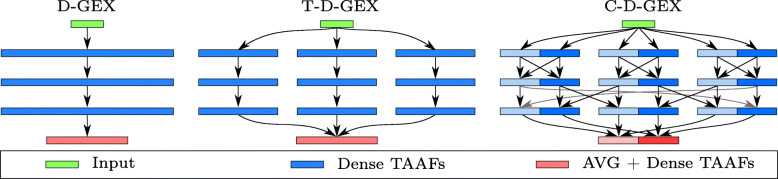
The original C–D–GEX architectures and the novel architectures proposed in this paper. The outputs of the towers (or halves for C–D–GEX) are averaged before the output layer

**Fig. 2:**
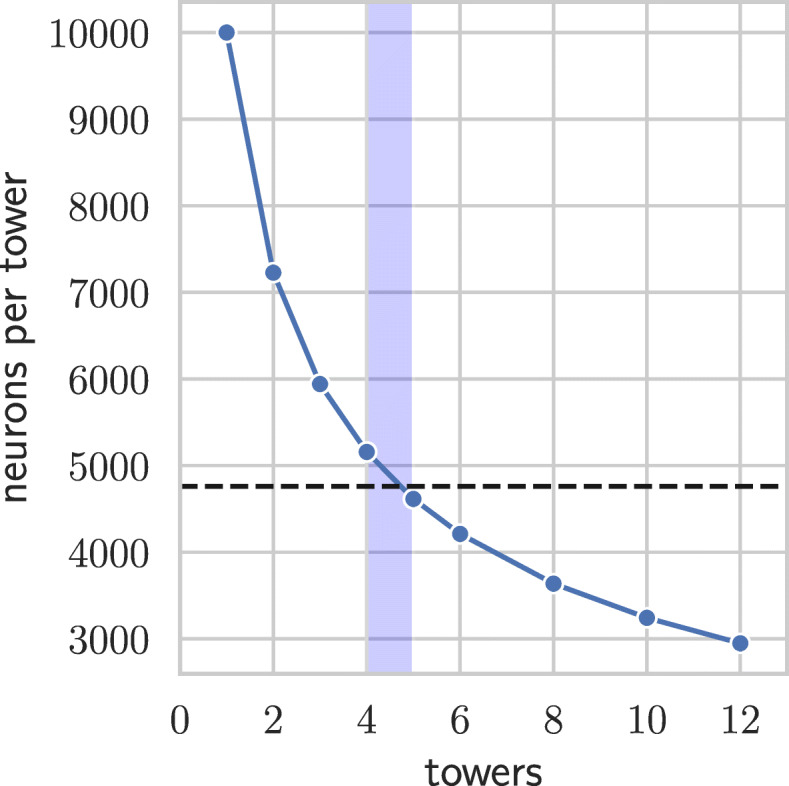
The relationship between neurons per tower and number of towers when the number of weights is limited by the number of weights of a single tower with 10,000 neurons. The dashed line represents the number of neurons in the output layer, the shaded region denotes the number of towers for which the number of neurons in each layer of each tower is the most similar to the number of output neurons

**Fig. 3:**
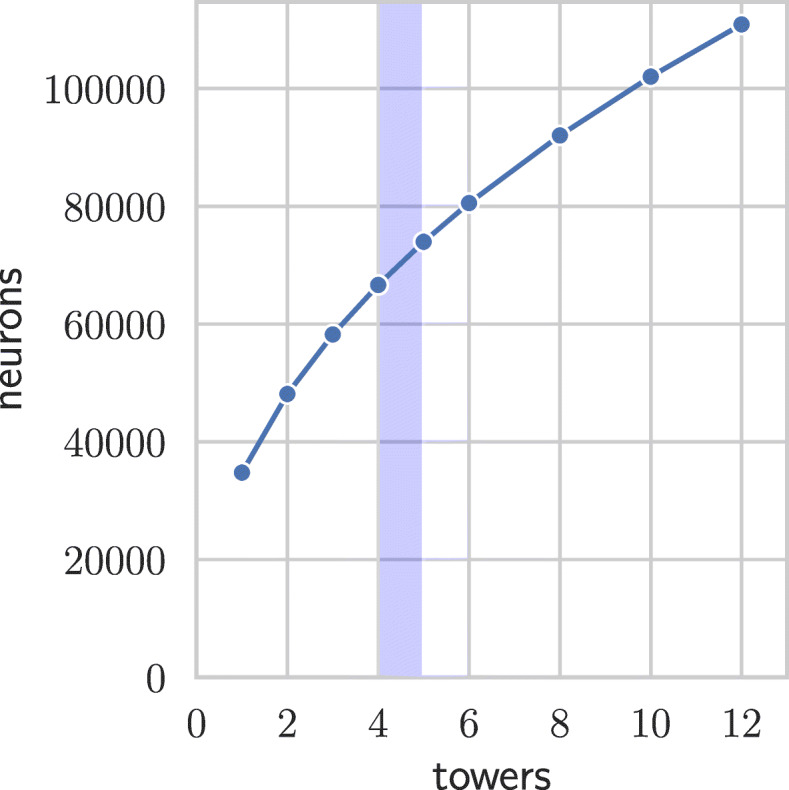
The relationship between the total number of neurons and number of towers when the number of weights is limited by the number of weights of a single tower with 10,000 neurons. The dashed line represents the number of neurons in the output layer, the shaded region denotes the number of towers for which the number of neurons in each layer of each tower is the most similar to the number of output neurons

Since the baseline model consists of three densely connected layers, a further increase in the number of neurons in each layer is difficult as the number of connections (weights) increases quadratically and even the baseline model was near the memory limitations of used GPU. Thus, similarly to PCs, we introduce towers of dense layers that are not connected to each other as depicted in Fig. 1, which allows for a significant increase in the number of neurons without the increase in the number of weights. Unlike the PCs [18, 19], the output layer is not densely connected to all the towers but rather the outputs of individual towers are first averaged and only then an output layer is added — otherwise the gains from the tower architecture would be much smaller as the number of connections between last hidden layer and the output layer would not change. The D–GEX with the tower architecture is denoted T–D–GEX, the number of neurons in a single layer of a tower was determined such that networks with more columns have strictly fewer weights (yet more neurons) as shown in Table 1 and Figs. 2 and 3.

**Table 1:** The summary of the parameterization of used architectures — C–D–GEX, CR–D–GEX, T–D–GEX, and TR–D–GEX do not differ in number of parameters and neurons. Note that the total number of parameter remains approximately the same across architectures

Towers	Neurons/tower	Neurons	Parameters
1	10,000	34,759	257,149,036
2	7,227	48,121	257,119,561
3	5,941	58,228	257,068,283
4	5,157	66,643	256,997,503
5	4,615	73,984	256,977,621
6	4,211	80,557	256,953,201
8	3,637	92,047	256,729,407
10	3,242	102,019	256,587,674
12	2,948	110,887	256,374,168

#### Checkerboard architecture (C–D–GEX)

The checkerboard architecture can be seen as an extension of the tower architecture. The tower architecture consists of towers of densely connected layers where each layer is connected to the layer that precedes it; there is no information flow between the towers — the towers share the input layer, and then their outputs are averaged before the output layer. The checkerboard architecture addresses this issue and divides each layer of a tower into halves — each half is connected to the same half of the same tower of the preceding layer; however, it is connected to the other half of the same tower only every odd layer while every even layer it is connected to the other half of the neighboring tower resulting in a checkerboard-like pattern of densely connected blocks. Both checkerboard architectures used in this paper have the first hidden layer without a dropout. The D–GEX with the checkerboard architecture is denoted C–D–GEX.

#### Skip connections

Another improvement was the addition of a skip connection in a ResNet-like manner [15] — we have added a residual skip connection from first to second hidden layer to each tower; the output of the first hidden layer is added to the output of second hidden layer before proceeding to the third hidden layer. Such networks are denoted TR–D–GEX and CR–D–GEX, respectively.

### Training

Following the procedure in [7, 8], a pair of D–GEX networks is trained; each network is trained for predicting expression levels of one half of target genes. A Nadam optimizer [21] was used for the training; optimizer specific parameters were *β*_1_=0.9,*β*_2_=0.999, and schedule decay was *η*=0.004. The training procedure took 600 epochs and the learning rate was set to 5×10^−4^ for epochs 1 – 400, 5×10^−5^ for epochs 401 – 475, 5×10^−6^ for epochs 476 –550, 5×10^−7^ for epochs 551 – 575, and 2.5×10^−7^ for epochs 576 – 600.

### Activation function

We are using the modified D–GEX with transformative adaptive activation functions (TAAFs) [8]. A TAAF is an adaptive activation function that introduces four parameters that are learned during training and that allow for translation and scaling of the inner activation function of the TAAF. More specifically, let *x*_*i*_ be individual inputs to a neuron and *w*_*i*_ their weights, *f* an inner activation function (e.g., a logistic sigmoid), then the output of a neuron with TAAF is:

1 $$ \alpha \cdot f\left(\beta\cdot \sum_{i=0}^{n}w_{i}x_{i} + \gamma\right) +\delta,  $$

where *α*,*β*,*γ*, and *δ* are separate parameters for each neuron that are trained together with weights *w*_*i*_ [8].

### Performance evaluation

Performance evaluation follows the same standards as in [8]. The main metric for evaluation of the model performance is denoted MMAE and it is the absolute error averaged over individual samples and genes. Let $\mathcal {S}$ be a set of evaluated samples, $\mathcal {G}$ a set of measured target genes, *y*(*g*,*s*) is the target expression of gene $g \in \mathcal {G}$ for sample $s \in \mathcal {S}$ and $\widehat {y(g, s)}_{m}$ the prediction of expression of the gene for the sample *s* by a model *m*, then MMAE_*m*_ is defined as:

2 $$ \textrm{MMAE}_{m} = \frac{1}{|\mathcal{S}|} \sum_{s \in \mathcal{S}} \frac{1}{|\mathcal{G}|} \sum_{g \in \mathcal{G}} \left|y(g, s) - \widehat{y(g, s)}_{m}\right|.  $$

### Implementation

The work was implemented in python 3, the neural networks were implemented using *keras* [22] and tensor manipulation framework *Tensorflow* [23]. Other used packages are *pandas* [24], and *NumPy* [25] for data manipulation and *seaborn* [26] and *matplotlib* [27] for visualizations.

## Results

**Fig. 4:**
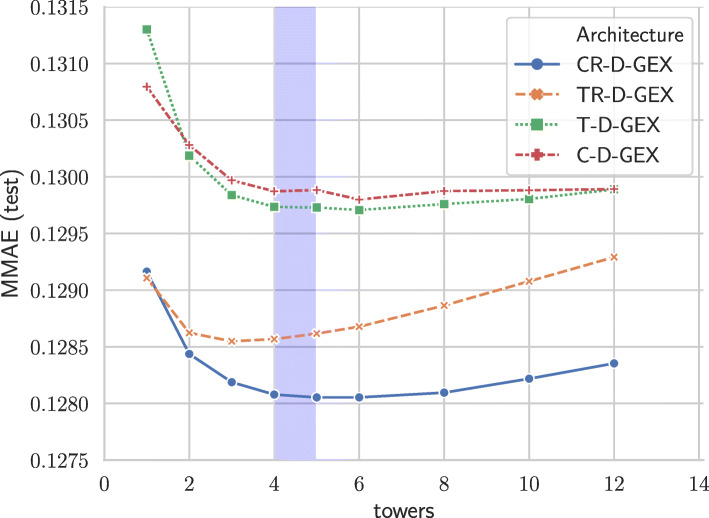
The development of MMAE based on the number of towers for individual architectures. The shaded region denotes the number of towers for which the number of neurons in each tower is the most similar to the number of output neurons

We have evaluated both modifications of the D–GEX architectures for nine different tower configurations. The configurations differ in the number of towers; their parameters are shown in Table 1 and the relationship between them is shown in Figs. 2 and 3. For each configuration, we have compared both possibilities for both configurations — tower or checkerboard architectures with or without the skip connection — resulting in a comparison of 2×2×9=36 different pairs of networks. The detailed results for all four architectures and different numbers of towers are shown in Table 2. The relationship of MMAE and the number of towers for different architectures is shown in Fig. 4 — we can observe that the MMAE drops quickly and then starts to rise again slowly. The drop in MMAE at the beginning is due to increase in the total number of neurons which then increases the capacity of the network making it able to better learn the relationships between the landmark and target genes. The relationship between MMAE and the number of towers seems to slightly differ across the architectures — e.g., the TR–D–GEX reaches the minimum MMAE for the lowest number of towers compared to other architectures — and thus further research is needed.

**Table 2:** The MMAE of tested models on the test data. Architecture similar to the D–GEX with TAAFs [8] is the T–D–GEX with 1 tower, the overall best model is shown in **bold**

# **Towers**	**T–D–GEX**	**C–D–GEX**	**TR–D–GEX**	**CR–D–GEX**
1	0.131301	0.130796	0.129107	0.129163
2	0.130187	0.130281	0.128623	0.128437
3	0.129839	0.129969	0.128548	0.128187
4	0.129735	0.129872	0.128568	0.128078
5	0.129729	0.129883	0.128617	**0.128053**
6	0.129707	0.129799	0.128677	0.128053
8	0.129760	0.129874	0.128864	0.128095
10	0.129804	0.129881	0.129078	0.128218
12	0.129889	0.129891	0.129291	0.128353
D–GEX (1×9,000, tanh) [7, 8]				0.163684
D–GEX with TAAFs (3×9,000,TAAFo sigmoid) [8]				0.134015

The networks with five or more towers actually introduce a bottleneck as the layers in individual towers contain fewer neurons than the output layer — the last layer has to infer the gene expression from a lower number of inputs than is the number of inferred genes. The number of towers for which the number of neurons is closest to the number of target genes is shown as a shaded region in Figs. 2, 3 and 4.

The T–D–GEX with one tower is the equivalent of the original D–GEX but with more neurons in each layer as the original D–GEX had at most 9,000 neurons in each layer and the tested architectures are based on T–D–GEX with 10,000 neurons. The increase in the number of neurons together with the learning rate schedule and increase in the number of training epochs led to the improvement of the single tower D–GEX’s MMAE from 0.134 to 0.131 even without the main architectural modifications. However, the proposed architectural changes, namely the CR–D–GEX (a checkerboard architecture with a skip connection from first to the second layer), led to MMAE of 0.128 without any increase in the number of parameters of the network and only a slight increase in the running time which is due to more neurons (more operations to be performed).

**Fig. 5:**
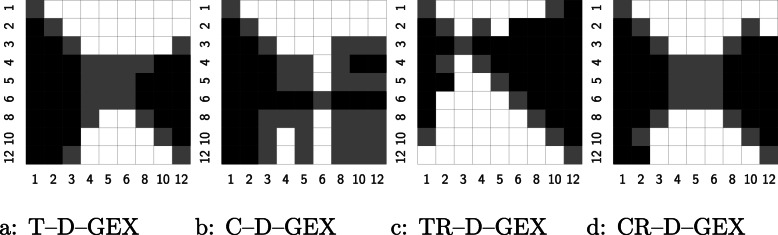
Results of pairwise Wilcoxon signed–rank test on the MAEs for individual samples for different number of towers. A cell in row *r* and column *c* is black if the model with *r* towers is statistically significantly better than model with *c* towers, white if worse, and grey if no statistically significant difference was observed

**Fig. 6:**
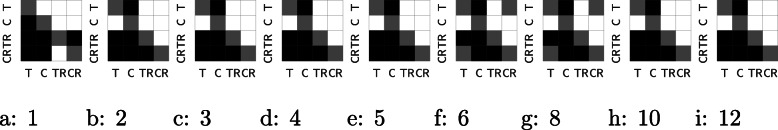
Results of pairwise Wilcoxon signed–rank test on the MAEs for individual samples for different architectures for fixed tower configuration. A cell in row *r* and column *c* is black if the model with architecture *r*-C–D–GEX is statistically significantly better than model with architecture *c*-D–GEX, white if worse, and grey if no statistically significant difference was observed

A Wilcoxon signed-rank test was used for pairwise comparison of individual models. The test was used to compare the means of MAEs of individual samples (which are assumed to be independent) at a significance level *α*=10^−4^. The results for comparing different tower configurations are shown in Fig. 5 and generally confirm the U-shape of the model performance shown in Fig.4. The comparison of different architectures for fixed tower configuration is shown in Fig. 6 — the checkerboard architecture CR–D–GEX is statistically significantly better for most configurations and not worse in all configuration with the exception of the single tower configuration. We have also compared the best architecture (CR–D–GEX with 5 towers) with the best D–GEX with TAAFs from [8] (3×9,000, TAAFo sigmoid) using the Wilcoxon signed-rank test and t-test on MAEs of individual samples and found that the CR–D–GEX has significantly lower MMAE with p–value <10^−6^. The 95% confidence interval for MMAE determined using bootstrap on samples’ MAEs with 10^5^ iterations was [0.12717,0.12891] for the CR–D–GEX with 5 towers and [0.13317,0.13487] for the D–GEX with TAAFs [8].

### Practical impact on differential gene expression analysis

**Fig. 7:**
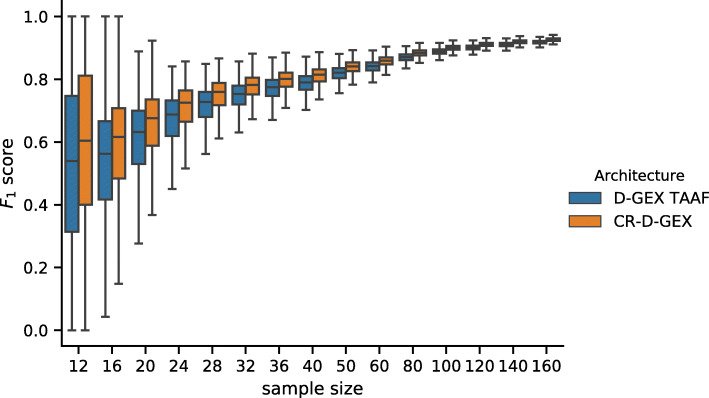
Distribution of the *F*_1_ scores obtained by the CR–D–GEX with 5 towers and the D–GEX with TAAF of 10,000 repetitions for each sample size. The whiskers show the 10^th^ and 90^th^ percentiles

**Fig. 8:**
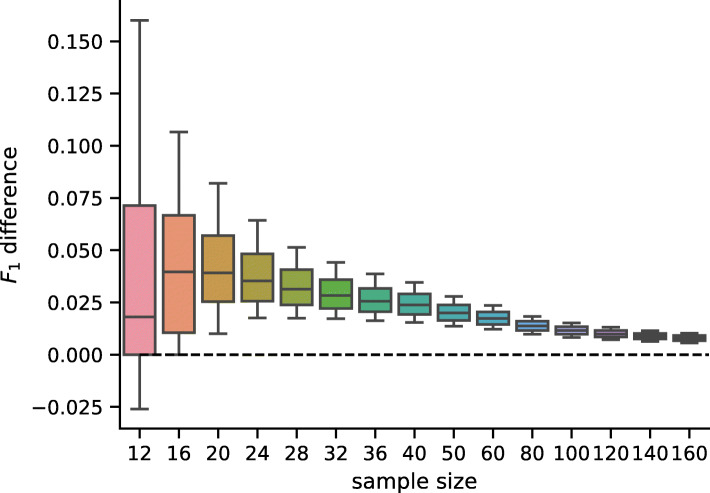
Distribution of pairwise differences of the *F*_1_ score obtained by the CR–D–GEX with 5 towers and the D–GEX with TAAF of 10,000 repetitions for each sample size. The whiskers show the 10^th^ and 90^th^ percentiles

**Fig. 9:**
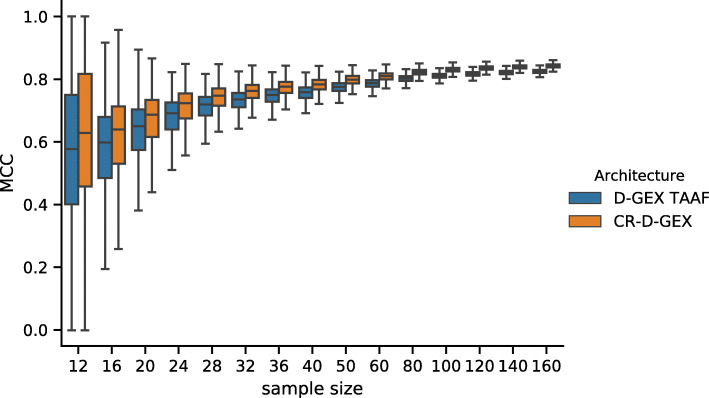
Distribution of the MCCs obtained by the CR–D–GEX with 5 towers and the D–GEX with TAAF of 10,000 repetitions for each sample size. The whiskers show the 10^th^ and 90^th^ percentiles

**Fig. 10:**
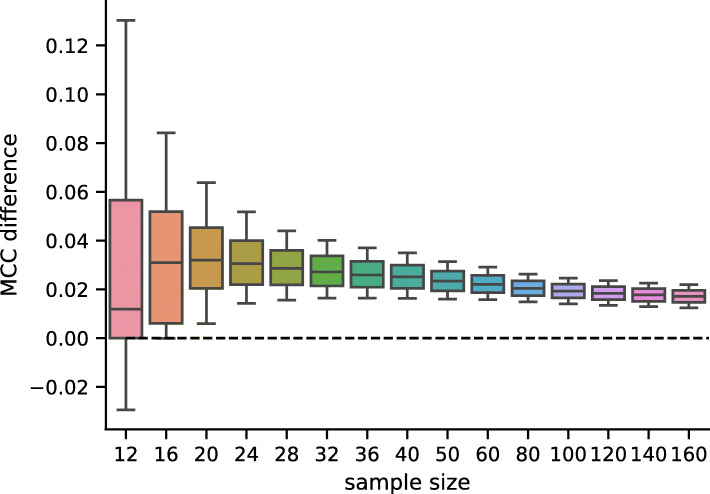
Distribution of pairwise differences of the MCCs obtained by the CR–D–GEX with 5 towers and the D–GEX with TAAF of 10,000 repetitions for each sample size. The whiskers show the 10^th^ and 90^th^ percentiles

While the checkerboard architecture has statistically significantly lower prediction error than the D–GEX with TAAFs [8], the practical impact of this improvement remains is unclear. We decided to demonstrate this impact on the frequent task of detection of differential gene expression. As there is no phenotype annotation available for the samples, we have generated it artificially. We have run hierarchical clustering on 2,000 samples sampled from the test data (i.e., unseen during the training of the model), then selected two large and relatively distinct clusters (containing 414 and 462 samples respectively). Then we have repeatedly sampled smaller datasets for different sample sizes (12–160) where each half of samples was from the same cluster and have run differential gene expression analysis using parametric empirical Bayes from the limma R package [28] on the ground truth data (the actual gene expression) and on the gene expressions inferred by the CR–D–GEX with 5 towers and the default D–GEX (TAAFo) [8]. For each model and each sampled dataset of a given size, we have calculated the F_1_ score of the prediction of the set of differentially expressed (DE) genes compared to the DE genes from the ground truth data found for the same sampled dataset. Then we have calculated the pairwise differences in the *F*_0.5_,*F*_1_,*F*_2_ scores, accuracy, and Matthews correlation coefficient (MCC) for both models. The distribution of values and the pairwise differences of F_1_ and MCC is shown in Figs. 7, 8, 9 and 10 for 10,000 repetitions for each sample size. The differences of all scores (*F*_0.5_,*F*_1_,*F*_2_ scores, accuracy, and MCC) were statistically significant for all tested sample sizes when using the Wilcoxon signed-rank test as all the p–values were <10^−8^. To provide interpretation for the observed pairwise differences in F_1_ score, the typical scenario was that CR–D–GEX with 5 towers reported much fewer false positive differentially expressed genes than TAAFo, sometimes at the cost of a very small increase in false negatives. Obviously, the advanced architectures can reasonably improve differential gene expression analysis and better approximate the gene sets reached with the original gene expression data. The improvement most strongly manifests for small sample sets, where even small changes in gene expression values may result in significant gene set changes.

## Discussion

The experimental results suggest that the introduction of Checkerboard and Tower architectures results in statistically significant improvement in the gene expression inference. At the same time, this improvement manifests in the practical gain when finding differentially expressed genes. We believe that this leads to new state-of-the-art results. Even though we are using a different normalization technique than other works [7, 29, 30], the relative increase in accuracy is larger than in the earlier results. Our reimplementation of the baseline D–GEX (without TAAFs) has MMAE 0.1637 while the best performing Checkerboard architecture has MMAE of 0.1284 ($1-\frac {0.1284}{0.1637}\approx 18\%$ improvement). The presented improvements using the more complex GAN approach have $1-\frac {0.2997}{0.3204}\approx 6.5\%$ improvement [30] and $1-\frac {0.2897}{0.3204}\approx 9.6\%$ in [29] over the baseline D-GEX. Moreover, both approaches (Checkerboards and GANs) are not mutually exclusive and can be potentially used together to reach even better performance. Furthermore, the Checkerboard architectures with TAAFs are conceptually much simpler than the usage of GANs while reaching, at the very least, comparable performance.

## Conclusion

Gene expression profiling was made cheaper by the usage of L1000 platforms and using computational techniques to infer the expression of the target genes. The quality of the inference was improved by the D–GEX [7] and its extension with TAAFs [8]. We have improved the architecture of D–GEX by introducing novel tower and checkerboard architectures and together with a small modification of the training parameters were able to improve the MMAE from 0.134 [8] to 0.128 (4.5% MMAE decrease). The improvement was tested using the Wilcoxon signed-rank test and t-test on the MAEs of individual samples and was found strongly statistically significant (p–value <10^−6^). We have also indirectly checked the practical significance of the improvement for the task of finding differentially expressed genes — our proposed model led to a statistically significant increase in the *F*_1_ score for different sample sizes (12 – 160). The best performing CR–D–GEX architecture allows better gene expression inference from the L1000 platform compared to the original CR–D–GEX. We have also hypothesized that the optimal number of towers for all tested architectures is such that the width of a layer in a tower is close to the width of the output layer and that the checkerboard architecture allows for lower error compared to the tower architecture while keeping the number of parameters constant.

### Future work

There are several directions in which this work will be expanded. Since the optimal number of towers for each architecture was different, one of the direction is to determine the relationship between the optimal number of columns and the used architecture. Other directions include a generalization of the checkerboard architecture — the checkerboard architecture divided each layer in each tower into halves and reconnected those in a certain pattern; however, dividing the layers into multiple folds and using more complex reconnection patterns might lead to networks with better performance and thus further improving the gene expression inference.

## Data Availability

The datasets analysed during the current study are available in the original D-GEX [7] repository, https://github.com/uci-cbcl/D-GEX.

## Footnotes

1 http://www.lincsproject.org/
